# Where to Stand with Stromal Cells and Chronic Synovitis in Rheumatoid Arthritis?

**DOI:** 10.3390/cells8101257

**Published:** 2019-10-15

**Authors:** Jean-Marc Brondello, Farida Djouad, Christian Jorgensen

**Affiliations:** 1 IRMB, Univ Montpellier, INSERM, F-34295 Montpellier, France; jean-marc.brondello@inserm.fr (J.-M.B.); farida.djouad@inserm.fr (F.D.); 2 CHU Montpellier, F-34295 Montpellier, France

**Keywords:** synovial, fibroblast-like synoviocytes, mesenchymal stem/stromal cells, rheumatoid arthritis, cellular identity, senescence

## Abstract

The synovium exercises its main function in joint homeostasis through the secretion of factors (such as lubricin and hyaluronic acid) that are critical for the joint lubrication and function. The main synovium cell components are fibroblast-like synoviocytes, mesenchymal stromal/stem cells and macrophage-like synovial cells. In the synovium, cells of mesenchymal origin modulate local inflammation and fibrosis, and interact with different fibroblast subtypes and with resident macrophages. In pathologic conditions, such as rheumatoid arthritis, fibroblast-like synoviocytes proliferate abnormally, recruit mesenchymal stem cells from subchondral bone marrow, and influence immune cell activity through epigenetic and metabolic adaptations. The resulting synovial hyperplasia leads to secondary cartilage destruction, joint swelling, and pain. In the present review, we summarize recent findings on the molecular signature and the roles of stromal cells during synovial pannus formation and rheumatoid arthritis progression.

## 1. Introduction

The joint synovial lining is a thin membrane divided into two anatomical and functional compartments: the surface layer (intima) and the sub-lining layer (subintima). The intima is a superficial layer of cells that produce lubricious synovial fluid in the intra-articular cavity. In healthy subjects, it is a thin membrane of two to three cell layers composed of fibroblast-like synoviocytes (FLS) and macrophage-like synovial cells.

Rheumatoid arthritis (RA) is an example of chronic disease that is primarily defined by joint synovial lining inflammation, resulting in subchondral bone and cartilage destruction. RA prevalence is 1–2% in Western countries. It is a multifactor autoimmune disease characterized by the presence of autoantibodies and genetic susceptibility [1,2]. Unlike osteoarthritis (OA), the most common age-related disease that affects frequently one single joint, RA is a systemic progressive joint disorder with early and late phases [3]. In patients with RA, the synovial membranes expand and their cellular component becomes highly heterogeneous. Specifically, the B and T cells that infiltrate the inflamed RA synovium, where they form aggregates, are qualitatively and quantitatively heterogeneous. The synovial resident CX3CR1^+^ macrophages form a dynamic immunological barrier in which macrophages are linked through tight junctions [4]. Conversely, monocyte-derived macrophages recruited from the circulation actively contribute to inflammation. In close contact, seven different fibroblast subpopulations are present in the RA synovium compared with OA [5]. In the present review, we will describe recent findings on synovium stromal cell heterogeneity during synovial pannus formation and RA progression.

## 2. Heterogeneity of Fibroblast-Like Synoviocytes in RA: A New Paradigm

According to the expression of surface markers, seven distinct FLS subpopulations have been described in RA synovium [5]. The podoplanin (PDPN), THY1 and cadherin-11 (CDH11)-positive, but CD34-negative fibroblast subset is expanded in patients with RA [5]. This CD34^−^PDPN^+^THY1^+^CDH11^+^ FLS subset has phenotypic characteristics of invasive cells and forms a perivascular zone surrounding capillary structures in the synovium sub-lining layer in contact with the lymphocytic infiltrate [5]. Moreover, gene expression analysis in CD34^−^THY1^+^ and CD34^+^ fibroblasts indicated that genes associated with fibroblast migration, such as *CTHRC1*, *TWIST1*, *POSTN*, *LOXL2*, *PDGFRB*, and *MMP14*, are up-regulated in these cell populations. Finally, upon stimulation with tumor necrosis factor (TNF), expression and secretion of chemokines, such as CXCL12, and CCL2, are increased in CD34^+^ FLS, suggesting a critical role in immune cell recruitment [5]. 

More recently, the existence of fibroblast subsets with non-overlapping functions have been described [6]. Indeed, FLS FAPα^+^THY1^+^ are associated with synovial inflammation, and express cytokines including IL6, IL33 and other chemokines that promote strong interactions with immune cells. Conversely, FAPα^+^THY1^−^ sub-lining FLS are associated with cartilage and bone erosion and increased expression of RANKL, MMP3, and MMP9. In a murine model of arthritis, specific depletion of FAPα^+^THY1^+^ FLS led to inflammation decrease, whereas depletion of THY1^−^ FLS resulted in bone protection, suggesting that these cell subtypes are putative therapeutic targets [6]. The number of FAPα^+^THY1^−^ cells was not significantly different in samples from patients with OA and RA, whereas that of PDPN^+^FAPα^+^THY1^+^ cells was strongly increased in RA samples. Finally, single-cell RNA sequencing of synovium-derived non-hematopoietic CD45^−^ cells identified five FLS subsets with specific gene signatures that underline their distinct pathological functions in RA. Specifically, genes associated with the formation of cartilage, bone and extra-cellular matrix (ECM) are overrepresented in the first FLS subset, whereas genes linked to inflammation are prevalent in the second subset. The third subset is enriched in genes involved in vasculogenesis and complement activation, and the fourth FLS population in genes that characterize highly proliferating populations. Finally, genes involved in hydrogen transport and acid secretion are upregulated in the fifth subset [6]. Thus, among the seven distinct FLS subpopulations identified in RA synovium that exhibit different phenotypes, at least two might display overlapping functions. 

Remarkably, RA FLS are less susceptible to contact inhibition, and are resistant to apoptosis [7]. This last feature favors synovial hyperplasia. RA FLS can migrate from joint to joint, and consequently have an important role in disease spreading (Table 1) [8,9]. Therefore, advances in understanding the biology of FLS, including their capacity to regulate the innate immune response, their migration potential and their invasive properties, provide novel insights into RA pathogenesis. 

In RA, inflammatory memory has been classically related to the immune system. However, recent studies have shown that the innate immune memory may be driven also by FLS. In RA, synovial-derived fibroblasts maintained an inflammatory response to TNFα up to four days after challenge [10]. Comparison of fibroblasts isolated from different body sites suggested that inflammatory memory is present in synovial and gingival fibroblasts, but not in dermal fibroblasts. Repeated challenges with toll-like receptor 2 (TLR2) or TLR4 induced a refractory state only in fibroblasts from some sites, such as synovium and tendon [11]. Intracellular signaling by tendon fibroblasts diminished over time after stimulus removal, although PDPN and vascular cell adhesion molecule 1 (VCAM-1) were strongly expressed, indicating persistent fibroblast activation. These findings suggest that fibroblast inflammatory memory can be a property linked to their site of origin rather than to the disease condition. 

## 3. Synovial-Derived Mesenchymal Stromal/Stem Cells: Where Do We Stand?

Pioneering studies to characterize synovial-derived Mesenchymal Stromal/stem Cells (MSC) were performed more than 14 years ago in our and other laboratories [17,18]. Based on the minimal criteria used at that time for defining MSC, we showed that although the differentiation capacities and transcriptomic signature of BM-derived and synovial-derived MSC are partially distinct, the two cell types share a similar phenotype ^17^ and meet the criteria defined by the Mesenchymal and Tissue Stem Cell Committee of the International Society for Cellular Therapy (ISCT) [19]. Other laboratories also claimed that multipotent progenitor cells, which they called MSC, are present in the synovium [20,21]. However, the origin of synovial MSC is still unknown.

Remarkably, the high chondrogenic potential of these “synovial-derived MSC” and the observation that articular cartilage, synovial sub-lining layer and ligaments originate from cells that express growth differentiation factor 5 (GDF5) suggest that cartilage and synovial membrane might have a common embryonic origin [22,23]. In agreement, cases of spontaneous synovial chondromatosis, a benign phenomenon characterized by synovial chondrogenesis, have been reported [24]. Thus, in the joint environment, synovial MSC with a strong chondrogenic potential, which were described several years ago, might correspond to the recently described FLS subset that expresses genes associated with the formation of cartilage, bone and ECM [6]. 

Where do these synovium-derived MSC come from? The RA synovium includes about 30% of multipotent cells that originate from BM (BM-MSC) [25]. The molecular mechanisms underlying BM-MSC recruitment to the synovium involve chemokines [26] and growth factors (e.g., placental growth factors, PlGF) that are abundantly produced in arthritic joints [27]. BM-MSC recruitment to the synovium promotes their interaction with resident FLS. This will further enhance PlGF production, and promote angiogenesis and chronic synovitis. Thus, indirectly, BM-MSC expand arthritis to healthy joints since arthritic synovial fibroblasts circulate [28]. This suggests that BM-MSC are implicated in RA initiation and progression, and to the direct recruitment and migration of other cell types, such as immune cells [28].

On the other hand, Sergijenko et al. have shown that synovial hyperplasia involves the infiltration of BM-derived cells, mainly CD16^+^CD32^+^ hematopoietic cells and a minority of MSC that express PDGFRA (a MSC/fibroblast marker), as well as the proliferation of synovium-derived MSC [29]. These two MSC populations are potentially functionally distinct subtypes. Moreover, it has been reported that another population of CD271^+^ MSC with a perivascular distribution and found in all synovial tissues expand in RA and OA. These cells have enhanced abilities to produce pro-inflammatory factors, indicating a pathogenic role in arthritis [30]. Altogether, these studies revealed the existence of different types of synovial MSC, possibly with redundant functions in RA that need to be thoroughly investigated.

In most mammals, spontaneous cartilage repair does not occur in vivo, although MSC are present in the synovium. Indeed, in humans, due to the large size of knee joints, synovial-derived MSC cannot migrate across large distances to reach the cartilage lesion. In the mouse, in response to a longitudinal full-thickness joint injury, synovial-derived MSC proliferate and differentiate into chondrocytes to form a cartilaginous extracellular matrix that expresses chondrocytic markers, such as SOX9 and type II collagen, within the synovium [31]. However, no cartilage repair was observed in this study. In contrast, in adult rabbits, when partial full-thickness defects were treated, MSC from the synovial membrane entirely filled the defect cavity to form a continuous layer of cells extending from the synovial membrane to the articular cartilage defect [32]. This suggests that cells of synovial origin can be mobilized for cartilage repair. 

## 4. Epigenetic Alterations in FLS and MSC during Rheumatoid Arthritis Progression

Epigenetic marks, such as DNA methylation and histone acetylation, are key molecular events that integrate environmental clues. They are ideal candidates to bridge the environmental and genetic contributions to RA risk. For instance, RA FLS have lost the capacity to repress inflammatory genes induced by TNFα, but they also promote inflammation independently of the inflammatory milieu of the RA joint [33]. Epigenetic analysis of isolated RA FLS shows histone H3K27 acetylation and increased chromatin accessibility in regulatory elements associated with the NF-κB pathway, interferon-regulatory factors (IRFs), and activating protein-1 (AP-1). These findings suggests that reduced transcriptional repression in FLS due to changes in histone acetylation and chromatin remodeling contributes to the chronic synovitis observed in RA [34]. Epigenome analysis of RA synoviocytes revealed clusters of regions associated with active enhancers and promoters of transcription factors. These genes were mainly associated with the immune response but also with unexpected pathways, such as the “Huntington’s Disease Signaling”, Wnt, and clathrin pathways [35]. It is worth noting that well-known epigenetic regulators such as HDAC or sirtuins have been involved in RA epigenome (see review [36]).

Interestingly, the localization of FLS with different gene expression patterns in proximal (shoulder, knee) and distal digital joints is epigenetically regulated. In particular, different HOX genes, which specify regional identity, are expressed in FLS isolated from proximal and distal joints, and this influences the expression of ECM remodeling genes [37]. For example, hand FLS express higher amounts of CXCL12, whereas shoulder FLS express higher levels of MMP1/MMP13. The PDPN and THY1 phenotype of FLS from proximal or distal joints is not known, but epigenetic alterations may explain the characteristic RA clinical phenotype with chronic synovitis mainly in distal joints (wrist, metacarpal phalangeal, and interphalangeal synovitis). This suggests that epigenetic alterations in FLS contributes to the chronic synovitis observed in RA as well as localization of the inflammatory joints.

## 5. Role of FLS in Immune Cell Recruitment and Retention

Synovial pannus formation is sustained mainly by proliferation of synovial fibroblasts and infiltration of immune cells. In RA FLS, CCL20 expression upregulation will promote the migration into the inflamed joints of Th17 cells [38]. Additionally, in the RA synovium, IL17-induced CCL20 production promotes the chemotaxis of monocytes, T cells, B cells and immature dendritic cells, either directly or indirectly in a CXCL12-dependent manner (for review see [39]). Perivascular CD34^−^THY1^+^ FLS from patients with RA overexpress chemokines (CXCL12, CCL2, and RANTES) and play a critical role in immune cell recruitment [40] phocytes and monocytes [41]. CCL2 promotes the recruitment of CCR2^+^ macrophages, T cells, and natural killer cells (for review see [42]). RANTES chemotactic role for lymphocytes in RA synovium was demonstrated in vitro [43]. FLS also express integrins (e.g., alpha4/beta1 integrins) and adhesion molecules (e.g., VCAM-1 and intercellular adhesion molecule 1) [44] that favor T cell adhesion and retention. Indeed, T cells, particularly CD4^+^ cells with the Th1 phenotype that strongly express CXCR3 and CCR5, are massively recruited within the inflamed joints where they have a pivotal role in joint inflammation and arthritis development [45,46]. Once recruited, T lymphocyte retention within the RA synovium is associated with enhanced adhesion of synovium-derived endothelial cells activated by TNF [47]. Moreover, the persistence of activated CD4^+^ T cells in the inflamed synovial membrane of patients with RA has been partly attributed to the increased expression of sphingosine kinase-1 by FLS that promotes the conversion of sphingosine into sphingosine-1-phosphate [48]. Of note, compared with OA, sphingosine-1-phosphate and its receptors are indeed strongly expressed in RA synovium [49,50]. This shows that FLS from patients with RA play a critical role in immune cell recruitment. 

## 6. Apoptotic and Proliferative Capacities of FLS and MSC in Rheumatoid Arthritis

FLS long-term survival potential is related to their low apoptosis rate and high proliferative capacities. Inhibition of apoptosis in RA FLS is related to changes in the expression of anti-apoptotic B-cell lymphoma-2 (BCL-2) and p53. BCL-2 expression in RA FLS is induced by inflammation, thereby protecting them from cell death. IL-15, a cytokine present in RA synovitis, also increases BCL-2 expression [51]. Remarkably, IL-15 blockade increases apoptosis in FLS through BCL-2 suppression. Expression of p53, an archetypical DNA-damage responsive tumor suppressive gene, also is elevated in RA FLS, but it is not required for p53 upregulated modulator of apoptosis (PUMA)-mediated apoptosis in these cells [52]. A knockout mouse model and RA FLS gene expression analyses demonstrated that RA FLS resistance to apoptotic signals can also be linked to reduced/loss of DICER1 expression [53]. DICER1 is an endonuclease involved in microRNA biogenesis and also in the DNA damage response and processing of cytotoxic non-coding RNA (review [54]). Finally, the co-localization of FLS expressing FAS receptors and immune cells positive for FAS ligand has been described [55]. FAS-induced apoptosis resistance depends on the PI3 kinase/AKT activity. While BID increased expression substantially increased FAS-induced apoptosis, its inhibition suppressed RA FLS apoptosis. Of note, the inhibition FLICE-inhibitory protein (FLIP) increases the apoptotic suppression of RA FLS in response to FAS ligand [56]. Altogether, these findings reveal a mixture of phenotypes that complicates the identification of therapeutic targets to eliminate by apoptosis disease-driven FLS in patients. 

## 7. Synovial Cell Metabolic Interplay in RA Joints

RA joints are characterized by excessive proliferation of stromal cells and the synovial infiltrate of innate and adaptive immune cells. This cell biomass increase imposes high energy demands and leads to acidification of the synovial environment, resulting in stromal cell metabolic adaptations. Recent studies confirmed the high glucose consumption and increased lactate concentration in synovial fluid samples from patients with RA (for review see [13,57]). Therefore, RA can be viewed also as an energy metabolism disorder in which FLS shift their metabolism towards anaerobic glycolysis [58]. Furthermore, increased ATP concentration in the extracellular joint microenvironment following inflammatory cues promotes FLS-mediated activation of the P2X7 purine receptor signaling pathway [59]. Finally, we recently demonstrated that BM-MSC exert their immunosuppressive functions on inflammatory T cells partly through cell-mediated mitochondrial transfer [15,60]. Indeed, when co-cultured with T lymphocytes and especially IL17-producing T lymphocytes, healthy BM-MSC can transfer their mitochondria to T cells, thus inducing their metabolic reprogramming toward oxidative phosphorylation. This active suppressive process is partially reduced in synovium-derived MSC isolated from patients with RA [15,60]. This finding argues for a role of mitochondrial transfer in the maintenance of RA chronic inflammation [15,60].

## 8. Linking Senescence Onset and Synovitis

Excessive proliferation and hyperplasia of stromal cells are driving forces in RA progression. Some studies suggest that they can be viewed as a tumor cell-like behavior of FLS. Intra-articular gene transfer of tumor-suppressive genes, such as the senescence-promoting p16^INK4a^ and p21^CDKN1A^ cell-cycle inhibitors, has been used in preclinical RA joint models with the aim of delaying/preventing these invasive and hyperproliferative stromal phenotypes [61,62,63,64]. Indeed, cell senescence is the strongest natural tumor suppressive mechanism that permanently blocks cell proliferation through cell-cycle inhibitor induction in response to intrinsic signals (telomere attrition or oxidative stress), but also following inappropriate extrinsic stimuli, such as chronic exposure to inflammatory cytokines or oncogenes [65]. Senescence results in cell metabolic adaptations, epigenetic modifications, resistance to apoptotic signals and a specific transcriptomic signature [66]. The senescence program will induce cellular functional changes [65], while promoting the establishment of a specific secretory phenotype, called a senescence-associated secretory phenotype (SASP) that allows the recruitment of immune cells to favor in vivo senescent cell clearance [67]. Thus, induction of senescence in FLS could have some therapeutic benefits in RA by blocking their proliferation and also by facilitating tissue clearance and repair by the immune system. However, some evidence suggests that senescence could contribute to RA progression by playing a negative role, as recently demonstrated in OA development [68,69]. Surprisingly, FLS, of which sub-groups need to be identified, harbor some features of senescent cells in response to RA chronic inflammation [66]. Indeed, as mentioned, apoptotic resistance, pro-inflammatory secretory profile, and DICER1 downregulation are well-known characteristics of senescent cells [54,65]. As occurs during neoplastic tissue transformation, where pre-cancerous cells acquire first a senescence phenotype before escaping cell-cycle arrest to become hyperproliferative [65], it seems that some sub-types of RA FLS could reach a senescence-like state during the early stages of RA, thus establishing a stromal inflammatory SASP that favors hyperproliferation of other stromal cells and immune cells. Furthermore, as RA is an autoimmune inflammatory disease, it is well established that T lymphocytes from RA patients become senescent upon chronic self-antigen exposure and express p16^INK4a^, p21^CDKN1a^ and CD57, but also accumulate DNA damage and telomere defects (for review [16,67]). The development of such oligoclonal senescent T cells with new functional properties, such as tissue invasiveness, is part of the early RA phenotype [16,70,71]. Remarkably, through the establishment of a specific Janus kinase (JAK)-dependent SASP, senescent T cells can also promote early osteoclastogenesis and bone loss during RA progression [72]. Thus, these senescent CD28^null^ CD57^+^ T cells become responsive to the synovial RA microenvironment during the early disease phase, consequently amplifying the chronic joint inflammation in the late phase [72]. All these findings suggest that it is important to thoroughly investigate senescence in FLS sub-types and CD4^+^ T cells in order to determine the consequence of their depletion in dedicated pre-clinical RA models. Indeed, senescent cell elimination with seno-suppressive drugs or by pharmaco-genetic tools has recently been used with great success in other chronic degenerative diseases (for review see [66,69]). Such future studies could lead to the development of innovative long-term therapies also for patients with RA [69].

## 9. Perspectives for Innovative Therapeutic Approaches

Stromal cells, such as FLS and MSC, are heterogeneous populations with a key role in immune memory, and in the recruitment of macrophages that influence immune cell metabolism. Epigenetic alteration in MSC and FLS may explain specific immunoregulatory effects according to localization in the body. Immunotherapies have largely proven their benefit to control cytokine-mediated inflammation. New therapies promoting the refractory state of FLS and of synovial stromal cells may prevent joint inflammation relapse. 

Many treatments currently used by clinicians for RA management rely on seno-therapeutic strategies that have been developed in the last 15 years to target chronic inflammation and reduce joint deterioration and pain in patients with autoimmune diseases. In particular, TNF inhibitors that target one of the main SASP actors are classical biological agents commonly used to treat RA. Remarkably, a recent study showed that such compounds act by reducing T lymphocyte replicative senescence and enhancing telomerase activities [73], demonstrating their anti-senescence effects. 

Moreover, small molecules targeting signaling pathways, such as the JAK pathway, are promising for RA treatment. Indeed, JAK1, JAK2 and JAK3 are required for SASP establishment at senescence onset. The JAK inhibitors tofacitinib and baricitinib are prescribed to patients with RA to circumvent chronic inflammation [74,75], further confirming the importance of senescence in RA. 

A better characterization of FLS epigenome alterations, metabolic regulations and immune memory will pave the way to new therapeutic strategies to prevent the chronicity of inflammation in RA.

## Figures and Tables

**Table 1:** Cell-type interactions and synovial pannus formation.

Cell Types	Inactive RA	Active RA Synovial Pannus Formation Chronic Non-Healing Wound Phenotype
Fibroblast-like synoviocytes (FLS)	Hyperplasia Potential autophagy defects [12]	Hyperplasia Interact with lymphoid-like structures Metabolic adaptation toward anaerobic glycolysis [13,14] Lactate export and micro-environment acidification leading to inflammation Increase in ATP sensors: P2X7 expression [13]
Mesenchymal stroma/stem cells (MSC)		Reduced capacities to transfer mitochondria [15] Migrate to synovial pannus
Immune cells (T, B, Plasma, Antigen-presenting cells)	Oligoclonal-induced senescence in T cells [16] Premature telomere uncapping in T cells [16]	Lymphoid-like structure Invasive phenotypes [16] Aberrant nutrition sensing [16]
Endothelial cells		Neovascularization Glycolysis [16]

## References

[1] Rudan I., Sidhu S., Papana A., Meng S.J., Xin-Wei Y., Wang W., Campbell-Page R.M., Demaio A.R., Nair H., Sridhar D. (2015). Prevalence of rheumatoid arthritis in low- and middle-income countries: A systematic review and analysis. J. Glob. Health.

[2] Vicente R., Noël D., Pers Y.M., Apparailly F., Jorgensen C. (2016). Deregulation and therapeutic potential of microRNAs in arthritic diseases. Nat. Rev. Rheumatol..

[3] Smolen J.S., Aletaha D., Barton A., Burmester G.R., Emery P., Firestein G.S., Kavanaugh A., McInnes I.B., Solomon D.H., Strand V. (2018). Rheumatoid arthritis. Nat. Rev. Dis. Primers.

[4] Culemann S., Grüneboom A., Nicolás-Ávila J., Weidner D., Lämmle K.F., Rothe T., Quintana J.A., Kirchner P., Krljanac B., Eberhardt M. (2019). Locally renewing resident synovial macrophages provide a protective barrier for the joint. Nature.

[5] Mizoguchi F., Slowikowski K., Wei K., Marshall J.L., Rao D.A., Chang S.K., Nguyen H.N., Noss E.H., Turner J.D., Earp B.E. (2018). Functionally distinct disease-associated fibroblast subsets in rheumatoid arthritis. Nat. Commun..

[6] Croft A.P., Campos J., Jansen K., Turner J.D., Marshall J., Attar M., Savary L., Wehmeyer C., Naylor A.J., Kemble S. (2019). Distinct fibroblast subsets drive inflammation and damage in arthritis. Nature.

[7] Neumann E., Lefevre S., Zimmermann B., Gay S., Muller-Ladner U. (2010). Rheumatoid arthritis progression mediated by activated synovial fibroblasts. Trends Mol. Med..

[8] Lefèvre S., Knedla A., Tennie C., Kampmann A., Wunrau C., Dinser R., Korb A., Schnäker E.M., Tarner I.H., Robbins P.D. (2009). Synovial fibroblasts spread rheumatoid arthritis to unaffected joints. Nat. Med..

[9] Marinova-Mutafchieva L., Williams R.O., Funa K., Maini R.N., Zvaifler N.J. (2002). Inflammation is preceded by tumor necrosis factor-dependent infiltration of mesenchymal cells in experimental arthritis. Arthritis Rheumatol..

[10] Lee J.C., Min H.J., Park H.J., Lee S., Seong S.C., Lee M.C. (2013). Synovial membrane-derived mesenchymal stem cells supported by platelet-rich plasma can repair osteochondral defects in a rabbit model. Arthroscopy.

[11] Crowley T., Buckley C.D., Clark A.R. (2018). Stroma: The forgotten cells of innate immune memory. Clin. Exp. Immunol..

[12] Yang Z., Goronzy J.J., Weyand C.M. (2014). The glycolytic enzyme PFKFB3/phosphofructokinase regulates autophagy. Autophagy.

[13] Weyand C.M., Goronzy J.J. (2017). Immunometabolism in early and late stages of rheumatoid arthritis. Nat. Rev. Rheumatol..

[14] Falconer J., Murphy A.N., Young S.P. (2018). Review: Synovial Cell Metabolism and Chronic Inflammation in Rheumatoid Arthritis. Arthritis Rheumatol..

[15] Patricia L.-C., Hernandez F.J., Djouad F., Luque-Campos N., Caceido A., Kremer S.C., Brondello J., Vignais M., Pène J., Jorgensen C. (2019). Mesenchymal stem cell repression of Th17 cells is triggered by mitochondrial transfer. Stem Cell Res. Ther..

[16] Li Y., Goronzy J.J., Weyand C.M. (2018). DNA damage, metabolism and aging in pro-inflammatory T cells: Rheumatoid arthritis as a model system. Exp. Gerontol..

[17] Djouad F., Bony C., Häupl T., Uzé G., Lahlou N., Louis-Plence P., Apparailly F., Canovas F., Rème T., Sany J. (2005). Transcriptional profiles discriminate bone marrow-derived and synovium-derived mesenchymal stem cells. Arthritis Res. Ther..

[18] De Bari C., Dell’Accio F., Tylzanowski P., Luyten F.P. (2001). Multipotent mesenchymal stem cells from adult human synovial membrane. Arthritis Rheumatol..

[19] Dominici M., le Blanc K., Mueller I., Slaper-Cortenbach I., Marini F., Krause D., Deans R., Keating A., Prockop D., Horwitz E. (2006). Minimal criteria for defining multipotent mesenchymal stromal cells. The International Society for Cellular Therapy position statement. Cytotherapy.

[20] De Bari C., Dell’Accio F., Luyten F.P. (2001). Human periosteum-derived cells maintain phenotypic stability and chondrogenic potential throughout expansion regardless of donor age. Arthritis Rheumatol..

[21] Sakaguchi Y., Sekiya I., Yagishita K., Muneta T. (2005). Comparison of human stem cells derived from various mesenchymal tissues: Superiority of synovium as a cell source. Arthritis Rheumatol..

[22] McGonagle D., Baboolal T.G., Jones E. (2017). Native joint-resident mesenchymal stem cells for cartilage repair in osteoarthritis. Nat. Rev. Rheumatol..

[23] Koyama E., Shibukawa Y., Nagayama M., Sugito H., Young B., Yuasa T., Okabe T., Ochiai T., Kamiya N., Rountree R.B. (2008). A distinct cohort of progenitor cells participates in synovial joint and articular cartilage formation during mouse limb skeletogenesis. Dev. Biol..

[24] Brabyn P.J., Capote A., Munoz-Guerra M.F., Zylberberg I., Rodriguez-Campo F.J., Naval-Gias L. (2018). Arthroscopic Management of Synovial Chondromatosis of the Temporomandibular Joint. Case Series and Systematic Review. J. Maxillofac. Oral Surg..

[25] Li X., Makarov S.S. (2006). An essential role of NF-kappaB in the “tumor-like” phenotype of arthritic synoviocytes. Proc. Natl. Acad. Sci. USA.

[26] Augello A., Kurth T.B., de Bari C. (2010). Mesenchymal stem cells: A perspective from in vitro cultures to in vivo migration and niches. Eur. Cell Mater..

[27] Yoo S.A., Park J.H., Hwang S.H., Oh S.M., Lee S., Cicatiello V., Rho S., de Falco S., Hwang D., Cho C.S. (2015). Placental growth factor-1 and -2 induce hyperplasia and invasiveness of primary rheumatoid synoviocytes. J. Immunol..

[28] Parsonage G., Filer A.D., Haworth O., Nash G.B., Rainger G.E., Salmon M., Buckley C.D. (2005). A stromal address code defined by fibroblasts. Trends Immunol..

[29] Sergijenko A., Roelofs A.J., Riemen A.H., de Bari C. (2016). Bone marrow contribution to synovial hyperplasia following joint surface injury. Arthritis Res. Ther..

[30] Del Rey M.J., Fare R., Usategui A., Canete J.D., Bravo B., Galindo M., Criado G., Pablos J.L. (2016). CD271(+) stromal cells expand in arthritic synovium and exhibit a proinflammatory phenotype. Arthritis Res. Ther..

[31] Kurth T.B., Dell’accio F., Crouch V., Augello A., Sharpe P.T., de Bari C. (2011). Functional mesenchymal stem cell niches in adult mouse knee joint synovium in vivo. Arthritis Rheumatol..

[32] Hunziker E.B., Rosenberg L.C. (1996). Repair of partial-thickness defects in articular cartilage: Cell recruitment from the synovial membrane. J. Bone Joint Surg. Am..

[33] Bottini N., Firestein G.S. (2013). Duality of fibroblast-like synoviocytes in RA: Passive responders and imprinted aggressors. Nat. Rev. Rheumatol..

[34] Loh C., Park S.H., Lee A., Yuan R., Ivashkiv L.B., Kalliolias G.D. (2019). TNF-induced inflammatory genes escape repression in fibroblast-like synoviocytes: Transcriptomic and epigenomic analysis. Ann. Rheum. Dis..

[35] Ai R., Laragione T., Hammaker D., Boyle D.L., Wildberg A., Maeshima K., Palescandolo E., Krishna V., Pocalyko D., Whitaker J.W. (2018). Comprehensive epigenetic landscape of rheumatoid arthritis fibroblast-like synoviocytes. Nat. Commun..

[36] Doody K.M., Bottini N. (2016). Chondrocyte clocks make cartilage time-sensitive material. J. Clin. Investig..

[37] Ospelt C., Frank-Bertoncelj M. (2017). Why location matters-site-specific factors in rheumatic diseases. Nat. Rev. Rheumatol..

[38] Hirota K., Yoshitomi H., Hashimoto M., Maeda S., Teradaira S., Sugimoto N., Yamaguchi T., Nomura T., Ito H., Nakamura T. (2007). Preferential recruitment of CCR6-expressing Th17 cells to inflamed joints via CCL20 in rheumatoid arthritis and its animal model. J. Exp. Med..

[39] Sarkar S., Fox D.A. (2010). Targeting IL-17 and Th17 cells in rheumatoid arthritis. Rheum. Dis. Clin. N. Am..

[40] Zhang F., Wei K., Slowikowski K., Fonseka C.Y., Rao D.A., Kelly S., Goodman S.M., Tabechian D., Hughes L.B., Salomon-Escoto K. (2019). Defining inflammatory cell states in rheumatoid arthritis joint synovial tissues by integrating single-cell transcriptomics and mass cytometry. Nat. Immunol..

[41] Oelkrug C., Ramage J.M. (2014). Enhancement of T cell recruitment and infiltration into tumours. Clin. Exp. Immunol..

[42] Mellado M., Martinez-Munoz L., Cascio G., Lucas P., Pablos J.L., Rodriguez-Frade J.M. (2015). T Cell Migration in Rheumatoid Arthritis. Front. Immunol..

[43] Shadidi K.R., Thompson K.M., Henriksen J.E., Natvig J.B., Aarvak T. (2002). Association of antigen specificity and migratory capacity of memory T cells in rheumatoid arthritis. Scand. J. Immunol..

[44] Bartok B., Firestein G.S. (2010). Fibroblast-like synoviocytes: Key effector cells in rheumatoid arthritis. Immunol. Rev..

[45] Mohan K., Issekutz T.B. (2007). Blockade of chemokine receptor CXCR3 inhibits T cell recruitment to inflamed joints and decreases the severity of adjuvant arthritis. J. Immunol..

[46] Wang C.R., Liu M.F. (2003). Regulation of CCR5 expression and MIP-1alpha production in CD4+ T cells from patients with rheumatoid arthritis. Clin. Exp. Immunol..

[47] Abbot S.E., Whish W.J., Jennison C., Blake D.R., Stevens C.R. (1999). Tumour necrosis factor alpha stimulated rheumatoid synovial microvascular endothelial cells exhibit increased shear rate dependent leucocyte adhesion in vitro. Ann. Rheum. Dis..

[48] Jaigirdar S.A., Benson R.A., Elmesmari A., Kurowska-Stolarska M.S., McInnes I.B., Garside P., MacLeod M.K.L. (2017). Sphingosine-1-Phosphate Promotes the Persistence of Activated CD4 T Cells in Inflamed Sites. Front. Immunol..

[49] Kitano M., Hla T., Sekiguchi M., Kawahito Y., Yoshimura R., Miyazawa K., Iwasaki T., Sano H., Saba J.D., Tam Y.Y. (2006). Sphingosine 1-phosphate/sphingosine 1-phosphate receptor 1 signaling in rheumatoid synovium: Regulation of synovial proliferation and inflammatory gene expression. Arthritis Rheumatol..

[50] Limaye V., Xia P., Hahn C., Smith M., Vadas M.A., Pitson S.M., Gamble J.R. (2009). Chronic increases in sphingosine kinase-1 activity induce a pro-inflammatory, pro-angiogenic phenotype in endothelial cells. Cell. Mol. Biol. Lett..

[51] Kurowska M., Rudnicka W., Kontny E., Janicka I., Chorazy M., Kowalczewski J., Ziolkowska M., Ferrari-Lacraz S., Strom T.B., Maslinski W. (2002). Fibroblast-like synoviocytes from rheumatoid arthritis patients express functional IL-15 receptor complex: Endogenous IL-15 in autocrine fashion enhances cell proliferation and expression of Bcl-x(L) and Bcl-2. J. Immunol..

[52] You X., Boyle D.L., Hammaker D., Firestein G.S. (2006). PUMA-mediated apoptosis in fibroblast-like synoviocytes does not require p53. Arthritis Res. Ther..

[53] Alsaleh G., Nehmar R., Blüml S., Schleiss C., Ostermann E., Dillenseger J.P., Sayeh A., Choquet P., Dembele D., Francois A. (2016). Reduced DICER1 Expression Bestows Rheumatoid Arthritis Synoviocytes Proinflammatory Properties and Resistance to Apoptotic Stimuli. Arthritis Rheumatol..

[54] De Cauwer A., Mariotte A., Sibilia J., Bahram S., Georgel P. (2018). DICER1: A Key Player in Rheumatoid Arthritis, at the Crossroads of Cellular Stress, Innate Immunity, and Chronic Inflammation in Aging. Front. Immunol..

[55] Garcia S., Liz M., Gomez-Reino J.J., Conde C. (2010). Akt activity protects rheumatoid synovial fibroblasts from Fas-induced apoptosis by inhibition of Bid cleavage. Arthritis Res. Ther..

[56] Palao G., Santiago B., Galindo M., Paya M., Ramirez J.C., Pablos J.L. (2004). Down-regulation of FLIP sensitizes rheumatoid synovial fibroblasts to Fas-mediated apoptosis. Arthritis Rheumatol..

[57] Yang X.Y., Zheng K.D., Lin K., Zheng G., Zou H., Wang J.M., Lin Y.Y., Chuka C.M., Ge R.S., Zhai W. (2015). Energy Metabolism Disorder as a Contributing Factor of Rheumatoid Arthritis: A Comparative Proteomic and Metabolomic Study. PLoS ONE.

[58] García-Prat L., Martínez-Vicente M., Perdiguero E., Ortet L., Rodríguez-Ubreva J., Rebollo E., Ruiz-Bonilla V., Gutarra S., Ballestar E., Serrano A.L. (2016). Autophagy maintains stemness by preventing senescence. Nature.

[59] Caporali F., Capecchi P.L., Gamberucci A., Lazzerini P.E., Pompella G., Natale M., Lorenzini S., Selvi E., Galeazzi M., Pasini F.L. (2008). Human rheumatoid synoviocytes express functional P2 × 7 receptors. J. Mol. Med. (Berl).

[60] Vignais M.L., Caicedo A., Brondello J.M., Jorgensen C. (2017). Cell Connections by Tunneling Nanotubes: Effects of Mitochondrial Trafficking on Target. Cell Metabolism, Homeostasis, and Response to Therapy. Stem. Cells Int..

[61] Nonomura Y., Nagasaka K., Hagiyama H., Sekine C., Nanki T., Tamamori-Adachi M., Miyasaka N., Kohsaka H. (2006). Direct modulation of rheumatoid inflammatory mediator expression in retinoblastoma protein-dependent and -independent pathways by cyclin-dependent kinase 4/6. Arthritis Rheumatol..

[62] Sekine C., Sugihara T., Miyake S., Hirai H., Yoshida M., Miyasaka N., Kohsaka H. (2008). Successful treatment of animal models of rheumatoid arthritis with small-molecule cyclin-dependent kinase inhibitors. J. Immunol..

[63] Nasu K., Kohsaka H., Nonomura Y., Terada Y., Ito H., Hirokawa K., Miyasaka N. (2000). Adenoviral transfer of cyclin-dependent kinase inhibitor genes suppresses collagen-induced arthritis in mice. J. Immunol..

[64] Taniguchi K., Kohsaka H., Inoue N., Terada Y., Ito H., Hirokawa K., Miyasaka N. (1999). Induction of the p16INK4a senescence gene as a new therapeutic strategy for the treatment of rheumatoid arthritis. Nat. Med..

[65] He S., Sharpless N.E. (2017). Senescence in Health and Disease. Cell.

[66] Childs B.G., Gluscevic M., Baker D.J., Laberge R.M., Marquess D., Dananberg J., van Deursen J.M. (2017). Senescent cells: An emerging target for diseases of ageing. Nat. Rev. Drug Discov..

[67] Vicente R., Mausset-Bonnefont A.L., Jorgensen C., Louis-Plence P., Brondello J.M. (2016). Cellular senescence impact on immune cell fate and function. Aging Cell.

[68] Jeon O.H., David N., Campisi J., Elisseeff J.H. (2018). Senescent cells and osteoarthritis: A painful connection. J. Clin. Investig..

[69] Tachikart Y., Malaise O., Mumme M., Jorgensen C., Brondello J.M. (2019). Seno-suppressive molecules as new therapeutic perspectives in rheumatic diseases. Biochem. Pharmacol..

[70] Colmegna I., Diaz-Borjon A., Fujii H., Schaefer L., Goronzy J.J., Weyand C.M. (2008). Defective proliferative capacity and accelerated telomeric loss of hematopoietic progenitor cells in rheumatoid arthritis. Arthritis Rheumatol..

[71] Hohensinner P.J., Goronzy J.J., Weyand C.M. (2011). Telomere dysfunction, autoimmunity and aging. Aging Dis..

[72] Goronzy J.J., Henel G., Sawai H., Singh K., Lee E.B., Pryshchep S., Weyand C.M. (2005). Costimulatory pathways in rheumatoid synovitis and T-cell senescence. Ann. N. Y. Acad. Sci..

[73] Parish S.T., Wu J.E., Effros R.B. (2009). Modulation of T lymphocyte replicative senescence via TNF-{alpha} inhibition: Role of caspase-3. J. Immunol..

[74] Fleischmann R., Kremer J., Cush J., Schulze-Koops H., Connell C.A., Bradley J.D., Gruben D., Wallenstein G.V., Zwillich S.H., Kanik K.S. (2012). Placebo-controlled trial of tofacitinib monotherapy in rheumatoid arthritis. N. Engl. J. Med..

[75] Fleischmann R., Schiff M., van der Heijde D., Ramos-Remus C., Spindler A., Stanislav M., Zerbini C.A., Gurbuz S., Dickson C., de Bono S. (2017). Baricitinib, Methotrexate, or Combination in Patients with Rheumatoid Arthritis and No or Limited Prior Disease-Modifying Antirheumatic Drug Treatment. Arthritis Rheumatol..

